# Multimodal molecular imaging evaluation for early diagnosis and prognosis of cholangiocarcinoma

**DOI:** 10.1186/s13244-021-01147-7

**Published:** 2022-01-20

**Authors:** Jiong Liu, Wen Xiu Ren, Jian Shu

**Affiliations:** 1 Department of Radiology, The Affiliated Hospital of Southwest Medical University, Luzhou, 646000 Sichuan People’s Republic of China; 2 Nuclear Medicine and Molecular Imaging Key Laboratory of Sichuan Province, No 25 Taiping St, Jiangyang District, Luzhou, 646000 Sichuan People’s Republic of China

**Keywords:** Cholangiocarcinoma, Molecular imaging, Nuclear medicine, Magnetic resonance imaging, Optical imaging

## Abstract

Cholangiocarcinoma (CCA) is an aggressive and lethal malignancy with limited therapeutic options. Despite recent advances in diagnostic imaging for CCA, the early diagnosis of CCA and evaluation of tumor invasion into the bile duct and its surrounding tissues remain challenging. Most patients with CCA are diagnosed at an advanced stage, at which treatment options are limited. Molecular imaging is a promising diagnostic method for noninvasive imaging of biological events at the cellular and molecular level in vivo. Molecular imaging plays a key role in the early diagnosis, staging, and treatment-related evaluation and management of cancer. This review will describe different methods for molecular imaging of CCA, including nuclear medicine, magnetic resonance imaging, optical imaging, and multimodal imaging. The main challenges and future directions in this field are also discussed.

## Key points

- Molecular imaging has higher sensitivity and specificity than conventional imaging for the diagnosis of cholangiocarcinoma.
- Different molecular imaging methods have specific advantages for cholangiocarcinoma diagnosis.
- Molecular imaging has great potential for finding targets, probe synthesis, and clinical applications in cholangiocarcinoma.

## Introduction

Cholangiocarcinoma (CCA) is a primary malignant tumor that occurs in intrahepatic and extrahepatic bile duct epithelial cells with high invasiveness and heterogeneity [1, 2]. In > 90% of cases, the histological type of CCA is sclerosing adenocarcinoma [3]. According to the anatomical location of the tumor, CCA can be divided into intrahepatic cholangiocarcinoma (ICCA), perihilar cholangiocarcinoma (PCCA), and distal cholangiocarcinoma (DCCA) [4–6]. The incidence of CCA is the highest in Asia, with more than 80 cases per 100,000 population [7]. Surgery is the most effective treatment for CCA. However, because the clinical symptoms of CCA are nonspecific and early diagnosis is difficult, most patients present in the terminal stage of cancer. Thus, CCA patients typically resort to palliative care and the overall 5-year survival rate of is < 10% [4, 8–11]. Therefore, it is essential to explore more effective diagnostic methods.

CCA is diagnosed via a combination of clinical symptoms, imaging manifestations, biochemical features, and histological examinations, and imaging plays a crucial role. US is the first and more common choice for screening CCA because it is inexpensive and simple to perform. However, US is difficult to assess the range of tumor invasive and is not efficient for detecting small invasive CCAs. Moreover, the accuracy of US varies according to tumor type, equipment quality, and operator experience [12, 13]. CT and MRI can characterize the mass and evaluate bile duct dilatation, vascular infiltration, and lymph node invasion to some extent. CT is considered the standard imaging modality for detecting CCA features and for staging. MRI is superior to CT for diagnosis and staging, and is comprises specific sequences such as diffusion-weighted imaging and MRCP. However, it lacks accuracy for the evaluation of tumor invasion along the bile duct. PET scan imaging is used to assess and evaluate distant metastasis. In contrast to CT morphological imaging, DWI and PET improve the diagnostic sensitivity and provide additional tumor information, including predicting the risk of tumor recurrence and prognosis [14–18]. However, mucinous CCAs can lead to false-negative results when using PET scanning. PTC and ERCP play a critical role in the management of PCCA. These techniques not only detect malignant biliary strictures, but can also be used to collect biliary brush samples for cytological and genetic evaluation. However, their application is limited by complications such as pancreatitis and bleeding caused by the invasive procedure [4, 19, 20]. In addition, the performance of imaging examinations varies according to the type of CCA. For ICCA, US is not accurate for differentiating it from HCC, especially in patients with cirrhosis. CT, MRI, and PET are relatively accurate for the diagnosis of ICCA. It has been reported that the sensitivity and specificity of PET for ICCA is 93% and 80%, respectively [21]. For PCCA and DCCA, the accuracy of US is 80–95% in DCCA patients, whereas PCCA is more difficult to identify [22, 23]. The sensitivity and specificity of MRI/MRCP for detecting PCCA are 88–89% and 75–85%, respectively, and CT are 75–79% and 79–80%, respectively [24, 25]. When PET is used to diagnose ECCA, the sensitivity and specificity are low at 55% and 33%, respectively [21].

Molecular imaging is an emerging discipline at the intersection of molecular biology and traditional medical imaging. It uses imaging methods to display specific molecules at the tissue, cellular, and subcellular levels. It can assess the changes at the molecular level in vivo, and perform qualitative and quantitative imaging studies on the biological behaviors of molecules. Molecular imaging provides a noninvasive, timely, and cost-effective method to study the fundamental behavior of organisms, thereby improving our understanding of diseases [26, 27]. Compared with traditional imaging techniques, molecular imaging can detect the specific histopathological changes at the cellular and molecular levels before the morphological changes of the disease take place [26]. This technology has the advantage of finding smaller lesions while simultaneously providing a basis for differential diagnosis and curative effect evaluation. This allows a more accurate diagnosis of the disease. Molecular imaging methods used in clinical and preclinical research in CCA include nuclear medicine imaging (PET and SPECT), MRI, optical imaging, and multimodal imaging. In the near future, it is expected that molecular imaging techniques will be used to study the pathogenesis of CCA in more detail to identify the key components of the onset stage, thereby providing an early and definitive diagnosis of CCA. In addition, it may help predict which high risk patients will develop the disease and provide information for the design of effective targeted therapy for any patient population. This article reviews clinical and preclinical studies on the application of different molecular imaging modalities in CCA over the past two decades. The aim of this study was to describe and discuss the role and characteristics of different molecular imaging methods for CCA diagnosis and research. It is expected to improve the accuracy of early diagnosis of CCA, establish the best treatment strategies, and ameliorate the quality of life and prognosis of patients.

## Nuclear medicine imaging

Nuclear medicine imaging is an imaging technology that shows physiological and pathological activity by detecting the metabolic processes of tracers with radionuclide. PET/CT is one of the earliest functional metabolic imaging methods used in the clinic. It has certain advantages for tumor diagnosis, differential diagnosis, and tumor monitoring. PET/CT tracers are synthesized by covalent connections of isotopes [28]. The tracer that has been used clinically and is the most commonly applied PET/CT tracer is fluorine-18 fluorodeoxyglucose(^18^F-FDG), a glucose analog that can be selectively absorbed by cells characterized by high glucose metabolism. In addition, there are some PET/CT tracers used in preclinical studies, such as those targeting COX-2, VEGF and CXCR4.

Several research groups have demonstrated that the sensitivity of ^18^F-FDG-PET in primary CCA is 90% or higher [29–31]. A retrospective study of 54 patients showed that the sensitivity, specificity, and accuracy of PET scanning for the diagnosis of CCA were 92.3%, 92.9%, and 92.6%, respectively [29]. Moon et al. analyzed 54 patients in a retrospective study and showed that the sensitivity and specificity of ^18^F-FDG-PET in the diagnosis of primary CCA is 89.1% and 87.5%, respectively [30]. Glucose uptake is related to the primary tumor location, size, and histopathological differentiation of CCA [30]. Multiple studies indicate that the sensitivity of ^18^F-FDG-PET is higher in intrahepatic CCA than in perihilar and extrahepatic lesions. A retrospective study of 62 patients performed by Corvera et al. showed that the sensitivity and specificity of ^18^F-FDG-PET differs significantly between suspected intrahepatic CCA (95% and 100%) and extrahepatic CCA (69.2% and 66.7%) [31]. The results of ^18^F-FDG-PET in the diagnosis of CCA are also related to the growth mode of the tumor. A study of 36 patients performed by Anderson et al. showed that the detection rate of nodular tumors is higher than that of invasive tumors, and the sensitivity for nodular morphology is 85%, whereas that for invasive morphology is 18% [32].

**Fig. 1:**
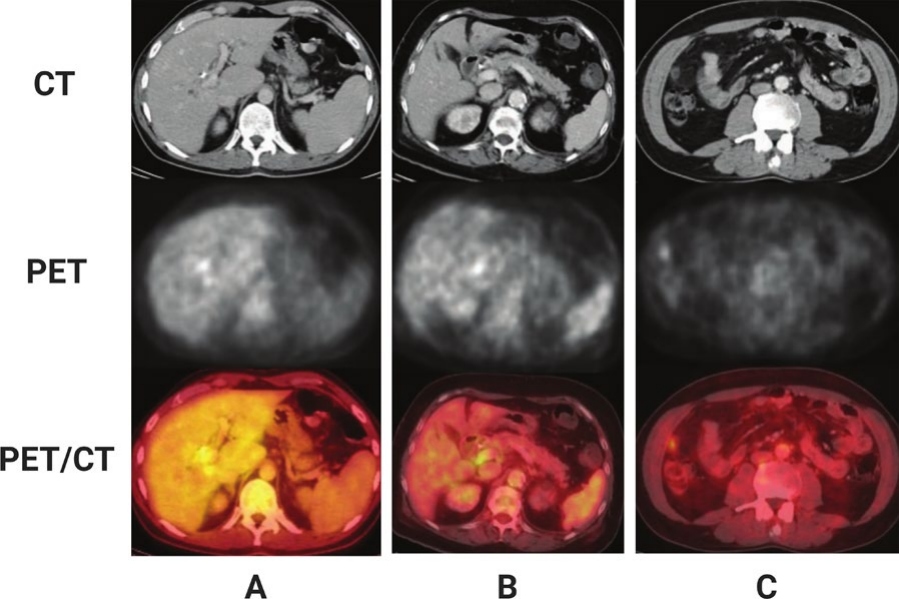
Three patients with perihilar cholangiocarcinoma. Reproduced from Li et al. [33]. **A** The hilar tumors on the CT scan also showed an increase in glucose metabolism on the PET scan. **B** CT analysis revealed that a lymph node along the head of the pancreas was enlarged. PET analysis showed that this region had high glucose metabolism. **C** CT analysis revealed a small nodule near the right abdominal wall with no obvious malignant features. However, PET/CT analysis showed that the nodule was a peritoneal metastasis, which was further verified by histopathology after surgical excision

Furthermore, the resectability of CCA is dependent on its local and distant spread. Li et al. used ^18^F-FDG-PET/CT to evaluate CCA prior to surgery. In that study, the operative and pathological results of 17 patients were reviewed for lymph node and distant metastasis (Fig. 1). The results showed that the sensitivity for the primary tumor was 58.8%, the sensitivity for lymph node and distant metastasis was 41.7–64.7% and 41.7–55.6%, respectively, and the specificity was 80–86.7% and 87.5–95%, respectively [33]. Another study indicated that the sensitivity of PET in detecting distant metastasis was only 65%; however, other lesions that were not detected by conventional imaging could be seen on PET, and their findings led to a change of treatment in 30% of patients with CCA [32]. Therefore, ^18^F-FDG-PET/CT can provide additional staging information for the preoperative diagnosis of lymph node and distant metastasis, which is a supplement to conventional CT scan.

**Fig. 2:**
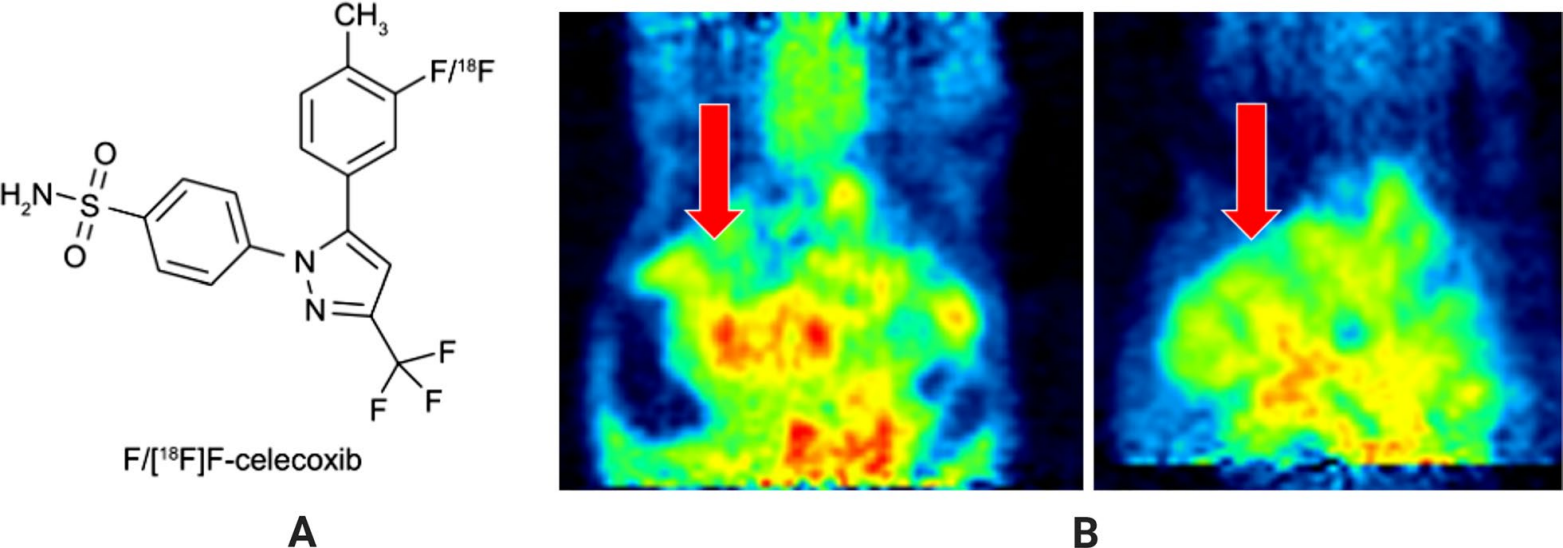
**A** Structure of ortho-[^18^F]F-celecoxib. **B** PET image for ortho-[^18^F]F-celecoxib was collected by scanning after caudal vein injection. Red arrow indicates the area for the liver of CCA rats (left) and normal rats (right), respectively. Reproduced from Chi-Wei et al. [35]

Local cholangitis and pericholangitis are related to the conversion of the biliary epithelium from atypical hyperplasia to malignant tumors [34]. Cyclooxygenase-2 (COX-2) plays a key role in this inflammatory cascade because it can catalyze the transformation of arachidonic acid to prostaglandins [35], which are inflammatory lipids that lead to local inflammation. Some human CCA cell lines express high levels of inducible COX-2 enzymes during inflammation [36]; therefore, COX-2 is considered a reasonable target for CCA. Chi-Wei et al. developed a PET imaging agent that could specifically target COX-2, ortho-[^18^F]F-celecoxib, which is synthesized by the addition of the radioactive atom ^18^F to the non-steroidal anti-inflammatory drug celecoxib (Fig. 2A). An investigation of ortho-[^18^F]F-celecoxib in rat CCA (Fig. 2B, C) showed that the amount of ortho-[^18^F]F-celecoxib uptake in CCA cells was significantly higher than that in normal cells. The ratio of tumor cells to normal cells was 1.38 ± 0.23, and the intake dose was 1.14 ± 0.25 (%ID/g) [35].

**Fig. 3:**
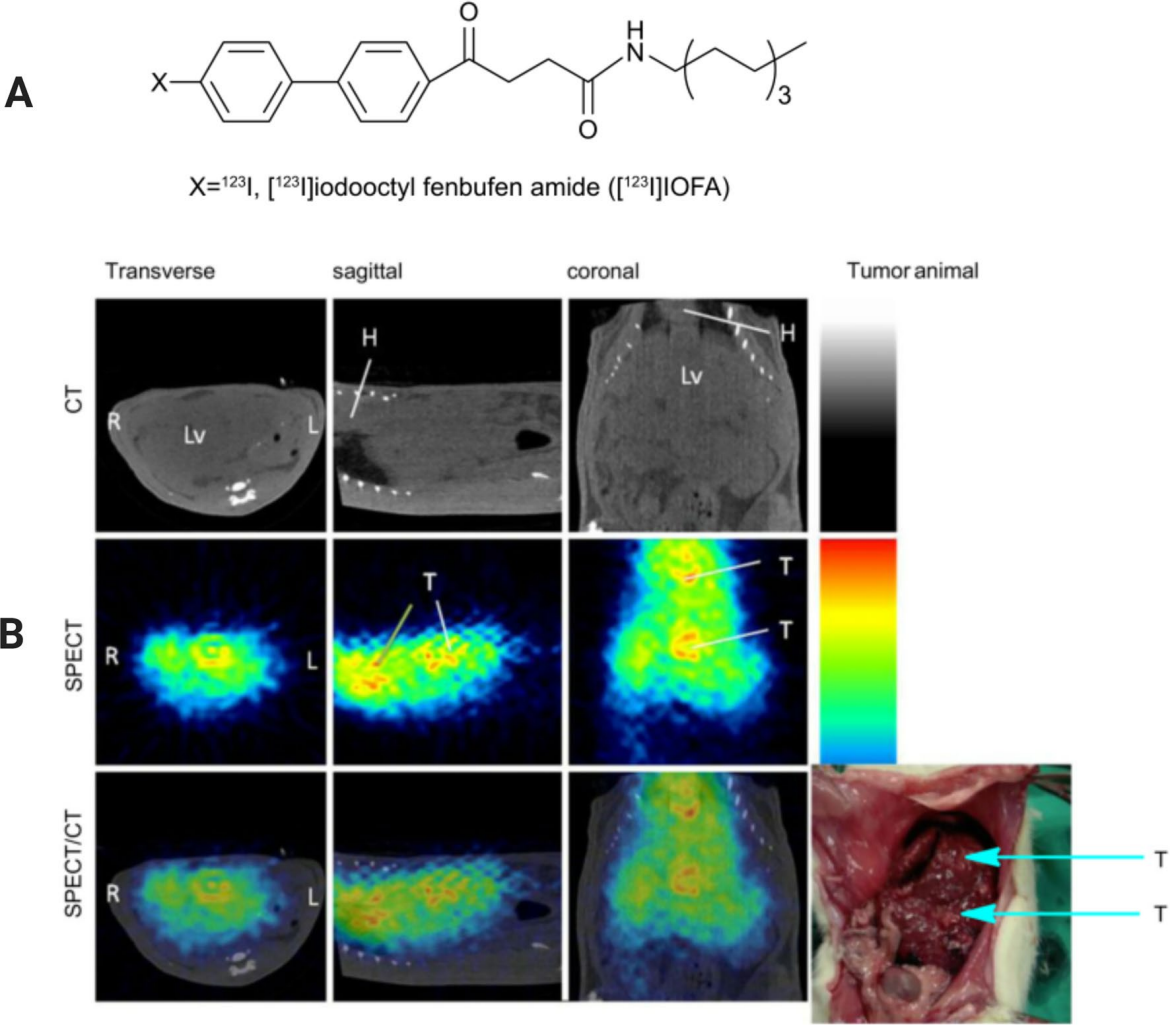
**A** Structure of radioiodine-labeled [^123^I]IOFA. **B** SPECT/CT images of CCA rats after injection of [^123^I]IOFA for 30–60 min.T: tumor, Lv: liver, H: heart. Reproduced from Huang et al. [37]

Huang et al. studied the effect of the SPECT reagent [^123^I]iodooctyl fenbufen amide ([^123^I]IOFA) on CCA in a similar way. The results showed that a lower level and homogeneous pattern of [^123^I]IOFA uptake were observed in the liver of normal rats. However, in the liver of rats with CCA, higher [^123^I]IOFA radioactivity absorption and heterogeneous patterns were regarded as hot spots of tumor lesions. An increase in COX-2 expression was detected by immunostaining in the bile ducts of CCA rats, but not in normal rats. Thus, the SPECT reagent [^123^I]IOFA has imaging potential for CCA with overexpression of COX-2 (Fig. 3) [37].

The inflammatory and stromal cells recruited by tumor cells release growth factors and chemokines, which stimulate the proliferation of tumor microvascular endothelial cells, thereby promoting tumor growth [38]. Vascular endothelial growth factor (VEGF) is a pleiotropic cytokine that binds to the extracellular domains of many different receptor kinases and participates in anti-apoptotic pathways, mitosis, and cell chemotaxis [36, 39]. Therefore, VEGF is the established target for anti-angiogenesis intervention. In recent years, receptor blocking antibodies and small molecule receptor kinase inhibitors has been developed as potential anti-angiogenic drugs. These molecules can attenuate VEGF-mediated signals, resulting in strong anti-proliferation and anti-angiogenic effects [6, 40]. CCA tumor cells also express high levels of VEGF, which leads to the production of a rich vascular bed. Li et al. labeled VEGF_165_ with ^123^I, and then used SPECT to image a variety of tumors, including CCA. In this study, four lesions from two CCA patients were included in the experimental observation. In the [^123^I]VEGF165 scan, three lesions showed increased uptake of imaging agent, and the detection rate of lesions was 75%. In this study, CT/MRI were superior to [^123^I]VEGF165 in displaying CCA, although [^123^I]VEGF165 may be a useful tool for visualizing CCA angiogenesis. Because the [^123^I]VEGF165 scan shows a cold spot in benign lesions, it is also helpful for the differential diagnosis of benign and malignant lesions and their activity.

**Fig. 4:**
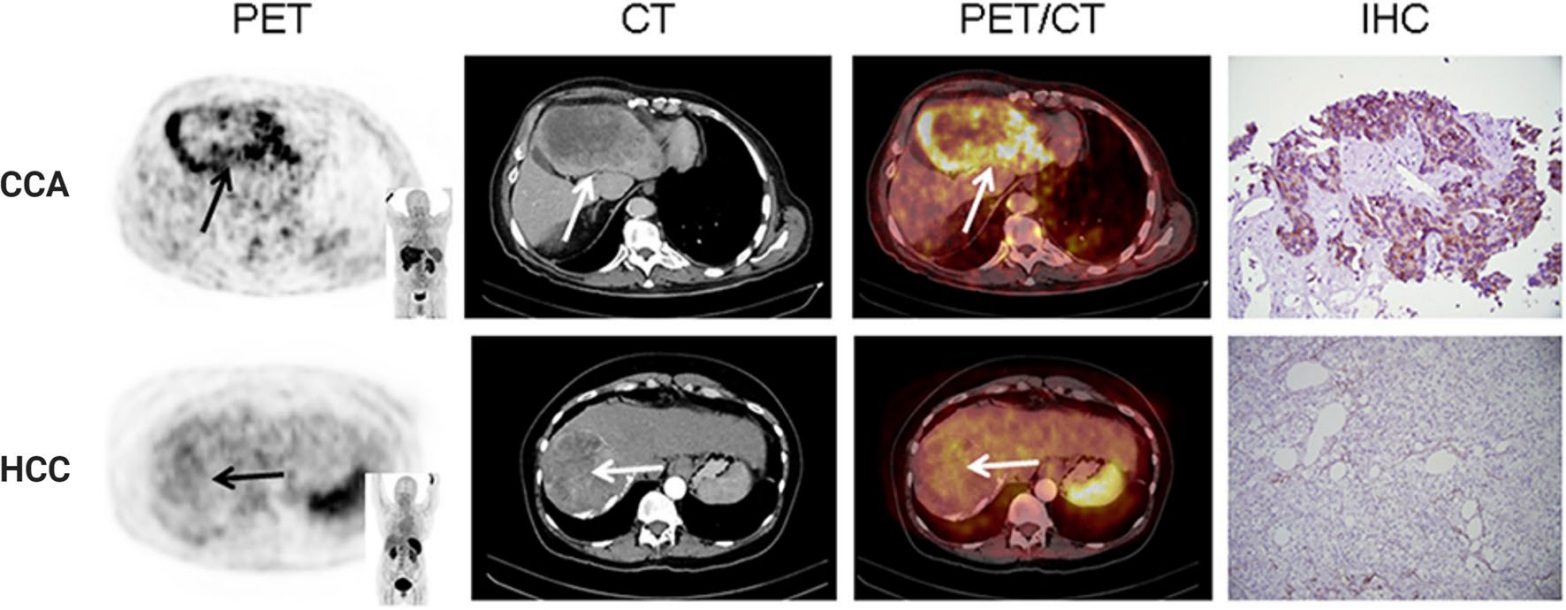
Results of immunohistochemical (IHC) and noninvasive CXCR4 cross-sectional PET, CT, and PET/CT fusion images in patients with cholangiocarcinoma and hepatocellular carcinoma (HCC). Cholangiocarcinoma showed high expression of CXCR4, while hepatocellular carcinoma showed no expression of CXCR4 on PET imaging. Reproduced from Werner et al. [47]

C-X-C motif chemokine receptor 4 (CXCR4) is highly expressed in more than 20 different types of tumors and plays an important role in tumor development, invasion, and metastasis, as well as cell-microenvironment interaction [41, 42]. A radiolabeled CXCR4 ligand, [^68^ Ga]Pentixafor, has high sensitivity and contrast in displaying the presence of receptors in vivo [43, 44]. PET imaging using [^68^ Ga]Pentixafor has been used in a variety of malignant tumors and inflammatory diseases [45, 46]. Werner et al. performed [^68^ Ga]Pentixafor PET/CT examinations on 19 newly diagnosed and untreated CCA patients along with other tumor groups, such as hepatocellular carcinoma (HCC), and found that the uptake level of radioactive tracer in CCA patients was the highest [47]. This result indicated the potential usefulness of CXCR4 as a target for CCA molecular imaging (Fig. 4) [47].

## MR imaging

Despite substantial research on traditional MRI and MRCP examination of CCA, there are few reports on MR molecular imaging of CCA. MR technology has significant advantages over other molecular imaging techniques, such as extremely fine spatial resolution, superior soft-tissue resolution, and no radiation. MR provides information regarding the change of tumor volume and the anatomical structure of the surrounding tissue while using the correlation between the increase in apparent diffusion coefficient (ADC) and tumor necrosis to quantitatively distinguish necrosis and tumor residue after treatment [48]. This makes MR an effective index to evaluate the efficacy of tumor treatments.

Compared with traditional gadolinium-based extracellular contrast agents, tissue-specific MR contrast agents targeting hepatobiliary or reticuloendothelial systems can increase the contrast between focus and liver, as well as the significance of focus on T1WI or T2/T2*WI after contrast. GD-EOB-DTPA is a gadolinium-based MR hepatobiliary-specific contrast agent. EOB-DTPA magnetic resonance cholangiography has high accuracy for the differential diagnosis of different subtypes of CCA [49]. A recent meta-analysis [50] showed that the sensitivity, specificity, and AUC of MRI extracellular contrast agents were 94%, 71%, and 0.92, respectively. This is comparable to the corresponding values for CT in evaluating the resectability of PCCA, although the use of EOB-DTPA improved the sensitivity and specificity. In addition, hepatobiliary contrast agent may not be suitable for CCA patients with cholestatic jaundice. Because cholestasis will decrease the uptake of contrast agent by hepatocytes, leading to an attenuation of degree of contrast [51]. In general, MRI combined with EOB-DTPA can accurately assess tumor scope, biliary tree, and vascular and adjacent structure involvement, in addition to facilitating differential diagnosis and providing prognostic information.

Superparamagnetic iron oxide (SPIO) consists of magnetic iron particles that can be specifically taken up by reticular endothelial cells. SPIO can magnify the nuclear magnetic resonance signal and improve the imaging sensitivity [52]. Few studies on SPIO for CCA have been reported, and the number of cases of CCA is rare in the known. Jin et al. conducted a comparative study of SPIO and mangafodipir for various liver diseases including three patients with CCA, and the results showed that the detection rate of SPIO for CCA was 100% [53]. In another study by Simone et al., a patient with CCA was successfully identified by blind evaluation using SPIO [54]. Polakova et al. indicated that oral SPIO negative contrast agent administered before MRCP improved the display rate of the extrahepatic bile duct, especially for patients with ascites [55].

SPIO has good surface activity, which allows it to interact with a variety of active substances to achieve active targeting [56]. A series of MR-specific probes were developed to improve SPIO targeting with good results. For example, Reichardt et al. confirmed that when small VEGF receptor tyrosine kinase inhibitors were combined with SPIO nanoparticles, the newly synthesized complex could be used to monitor the early response of adenocarcinoma to anti-angiogenic therapy through steady-state MR imaging [57]. Although some of these studies did not focus on CCA, certain aspects were similar. Thus, it is reasonable to believe that MR molecular imaging has great potential for future research on CCA.

Current MRI molecular imaging of CCA faces many challenges. First, part of MR contrast agents may have uncertain toxic effects to human body. Finding a contrast medium with good imaging effects, and no toxicity is difficult. Second, it is difficult for MRI to accurately evaluate CCA patients after biliary stent implantation. Lastly, the reliability of biomarkers of MRI molecular imaging remains uncertain and further research is urgently needed (Table 1).

**Table 1:** Characterization of traditional imaging techniques for CCA

Technique	Advantages	Limitations	Leading role
US	Inexpensive and simple to conduct	1. Difficulty in differential diagnosis 2. Difficult to assess the range of tumor invasive	First choice for screening
Enhanced CT	1. The sensitivity, specificity and accuracy in the evaluation of primary tumor, vascular and distant metastasis are very high 2. High spatial resolution	1. Radiation 2. Difficult to evaluate longitudinal invasion along the bile duct	Standard imaging mode for CCA diagnosis and staging
MRI/MRCP	1. Comprehensive evaluation of tumor, vascular and bile duct 2. No radiation 3. Multi-plane and multi-parameter imaging 4. Extremely high soft tissue resolution 5. Biliary tree visualization (MRCP)	1. Expensive cost 2. Long inspection time 3. Easy to be disturbed by artifacts	1. Differential diagnosis of difficult cases of CCA (except enhanced CT) 2. Evaluation of longitudinal invasion of ECCA along bile duct
ERCP	1. Evaluate bile duct strictures and intraluminal lesions 2. Both Diagnosis and treatment are feasible	1. Invasive complications 2. Difficult to evaluate the bile duct above the site of obstruction	Pathological diagnosis and biliary drainage
PET	1. Whole-body imaging 2. Extremely sensitive	May lead false positives and false negatives	Determination of distant Metastasis and tumor staging

US, ultrasound; MRCP, MR cholangiopancreatography; ECCA, extrahepatic cholangiocarcinoma; ERCP, endoscopic retrograde cholangiopancreatography

## Optical imaging

Optical imaging is gradually becoming a part of modern clinical medical imaging. Optical molecular imaging is based on the detection of optical information passing through biological tissues. The introduction of a suitable fluorescent probe allows detection of a fluorescence signal after excitation by a laser source of a specific wavelength. Alternatively, it can also introduce reporter genes, the products of which can fluoresce spontaneously. The emitted fluorescence carries tissue biochemical information related to absorption and scattering. The primary methods include bioluminescence imaging (BLI) and fluorescent imaging (FLI). BLI uses luciferase to label target cells or genes and their products, whereas FLI technology depends on cells or gene vectors carrying fluorescent reporter groups [58, 59]. Optical imaging has high sensitivity and superior spatial resolution similar to nuclear medical imaging, and the cost is relatively low. In addition, diffuse optical tomography (DOT) and fluorescent molecular tomography (FMT) can provide 3D optical information and have better depth sensitivity [60]. At present, most of the optical imaging studies applied to CCA are in the animal experimental stage, and only probe-based confocal laser endomicroscopy (pCLE) technology is used in the clinic. The pCLE combines optical imaging with confocal laser microendoscopy. It can be used to evaluate the subepithelial bile duct mucosa in vivo, and the additional microscopic information it provides is a promising diagnostic tool [61]. High-quality cross-sectional images of the epithelium will enable the characterization of tumors in vivo without multiple resections and biopsies in the near future [62, 63].

**Fig. 5.**
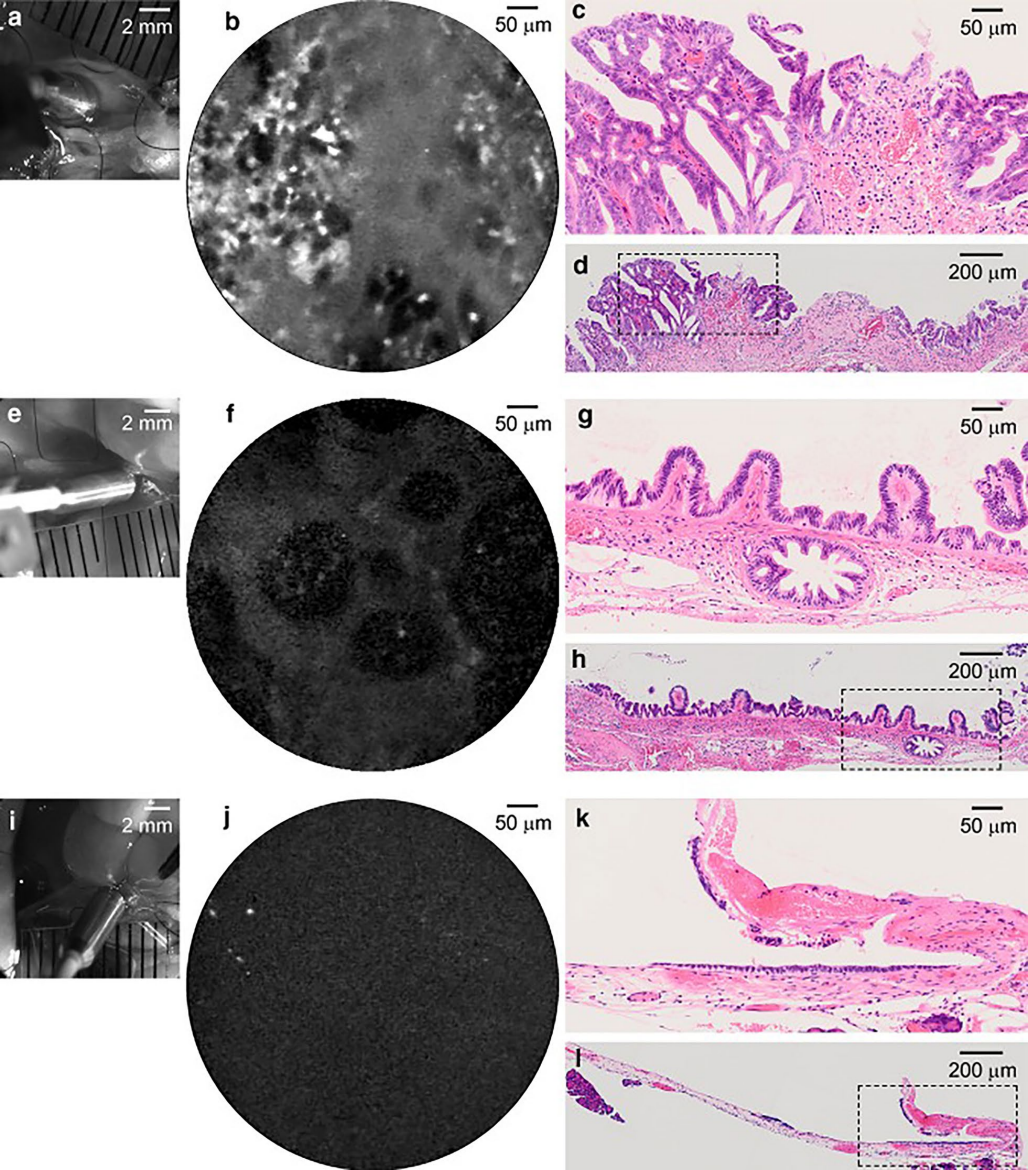
2-NBDLG fluorescence imaging of the hamster biliary tract by probe-based confocal laser endomicroscopy and matching histopathology sections. **a** Macro-zoom micrograph of 2-NBDLG fluorescence imaging process. **b** Fluorescence images after local injection of 2-NBDLG into the biliary tract. **c**, **d** HE staining of the corresponding sections in different scope fields. **e**–**h** Different animal in the same experimental group. **i**–**l** Normal control group. Reproduced from Hiroshi Yokoyama et al. [62]

The glucose transporter (GLUT) is a carrier that transports glucose across the mammalian cell membrane. Although the GLUT protein is not expressed in normal or benign lesions, it is expressed at high levels in malignant tumors [64]. 2-[*N*-(7-nitrobenz-2-oxa-1,3-diazol-4-yl) amino]-2-deoxy-d-glucose (2-NBDG) is a fluorescent tracer that enters living mammalian cells via GLUT in a time-, concentration-, and temperature-dependent manner. The fluorescence intensity of cells expressing GLUT increases significantly after exposure to 2-NBDG, and the cells can be distinguished more clearly [65]. However, its application to the diagnosis of cancer in vivo needs to be performed with caution because it is toxic to normal cells. Whether the fluorescence is emitted from tumor cells or non-tumor cells needs to be determined [66]. Yokoyama et al. found that 2-NBDLG, an l-glucose fluorescent derivative used as a functional probe for pCLE, could effectively reduce the background uptake of normal biliary tract cells and minimize the potential toxicity, thereby improving the imaging of CCA tumor cells (Fig. 5) [62].

FLI, BLI, DOT, and FMT also use the principle of optics for imaging. But, photon scattering and the absorption still limit the depth at which they can be used. Photoacoustic (PA) imaging was developed to solve the problem of imaging depth. Because the attenuation of the ultrasonic wave is three orders of magnitude smaller than that of photons, the imaging depth can be extended by several centimeters [60]. In addition, PA imaging has a unique property in that signals can be generated through endogenous luminescent groups in biological tissues (such as hemoglobin, myoglobin, lipids, and melanin). As a result, many biological processes in the body can be monitored, such as angiogenesis during tumor formation, the development of hypoxia in the tumor, and the visualization of blood flow in the tissue [67]. Zhang et al. designed a cystine knot peptide probe complex targeting integrin αvβ6 with high affinity for PA imaging of tumors [67]. Integrin αvβ6 is an important cell surface adhesion factor related to tumor invasion and metastasis. It is highly expressed in various malignant tumor cells, including CCA, but it is not expressed in normal adult tissues. Integrin αvβ6 shows potential for PA imaging of CCA.

Optical molecular imaging technology is an important tool for the study of small animal models, which provides unique insights into the pathogenesis of diseases, drug development, and therapeutic effects. Although optical molecular imaging is still in the preclinical cellular and small animal research and application stages, the development of molecular contrast agents that can be applied to patients is expected to expand the clinical applications of optical molecular imaging (such as endoscopy, intraoperative imaging, etc.). Compared with MRI, CT, and PET, optical imaging has several advantages such as the absence of electromagnetic radiation, high spatial resolution, real-time imaging ability, and large field of view, as well as low-cost and mobile imaging instruments. Although the lack of penetration depth due to tissue scattering and absorption of light hinders its use in whole-body imaging, optical molecular imaging provides a safe, real-time, non-invasive method for tumor detection and intraoperative imaging guidance, and it can depict the edge of the tumor and reveal cellular and molecular functional information in cancer. Therefore, the low depth of penetration should not hinder the development of optical molecular imaging methods.

## Multimodal imaging

Single imaging methods are associated with certain limitations. Nuclear medicine has extremely high sensitivity but poor spatial resolution, and it is thus not effective for locating the exact position of lesions. MR, however, has high spatial resolution but relatively poor sensitivity compared with nuclear medicine and optical imaging. Optical imaging has good sensitivity and spatial resolution, but the imaging depth is limited (Table 2). To overcome the limitations of a single imaging method, modern molecular imaging integrates different imaging components into one probe. This enables the acquisition of accurate anatomical, functional, or metabolic signals at the same time. For instance, the complementary combination of optical imaging and MRI can be applied to the examination of many diseases, and both are free of radiation. Therefore, research focusing on multimodal imaging has become more prominent. PET/MRI, SPECT/MRI, MRI/UCL (upconversion luminescent), and other dual-mode imaging methods have been successfully applied to animal models in vivo.

**Table 2:** Characterization of various molecular imaging modalities with CCA

Technique		Spatial resolution	Sensitivity	Depth	Time	Significant advantages
Nuclear	PET	6–10 mm	pM	Unrestricted	min-h	High sensitivity to deep tissue
	SPECT	7–15 mm	nM–pM	Unrestricted	min-h	
MR		1–1.5 mm	µM	Unrestricted	min-h	High spatial resolution to deep soft tissue and no radiation
Optical	BLI	0.1–2^a^ mm	fM	Few mm	min	High spatial resolution and sensitivity to superficial tissues
	FLI	0.1–2^a^ mm	pM	Few mm	min	
	FMT	1 mm	pM	1–2 cm	min	3D optical imaging and better depth sensitivity
	OA	0.01–1^a^ mm	pM	Several cm	min	Higher imaging depth than OI and can monitor biological process

µM, micromolar; nM, nanomolar; pM, picomolar; fM, femtomolar

^a^Spatial resolution is depth dependent

**Fig. 6:**
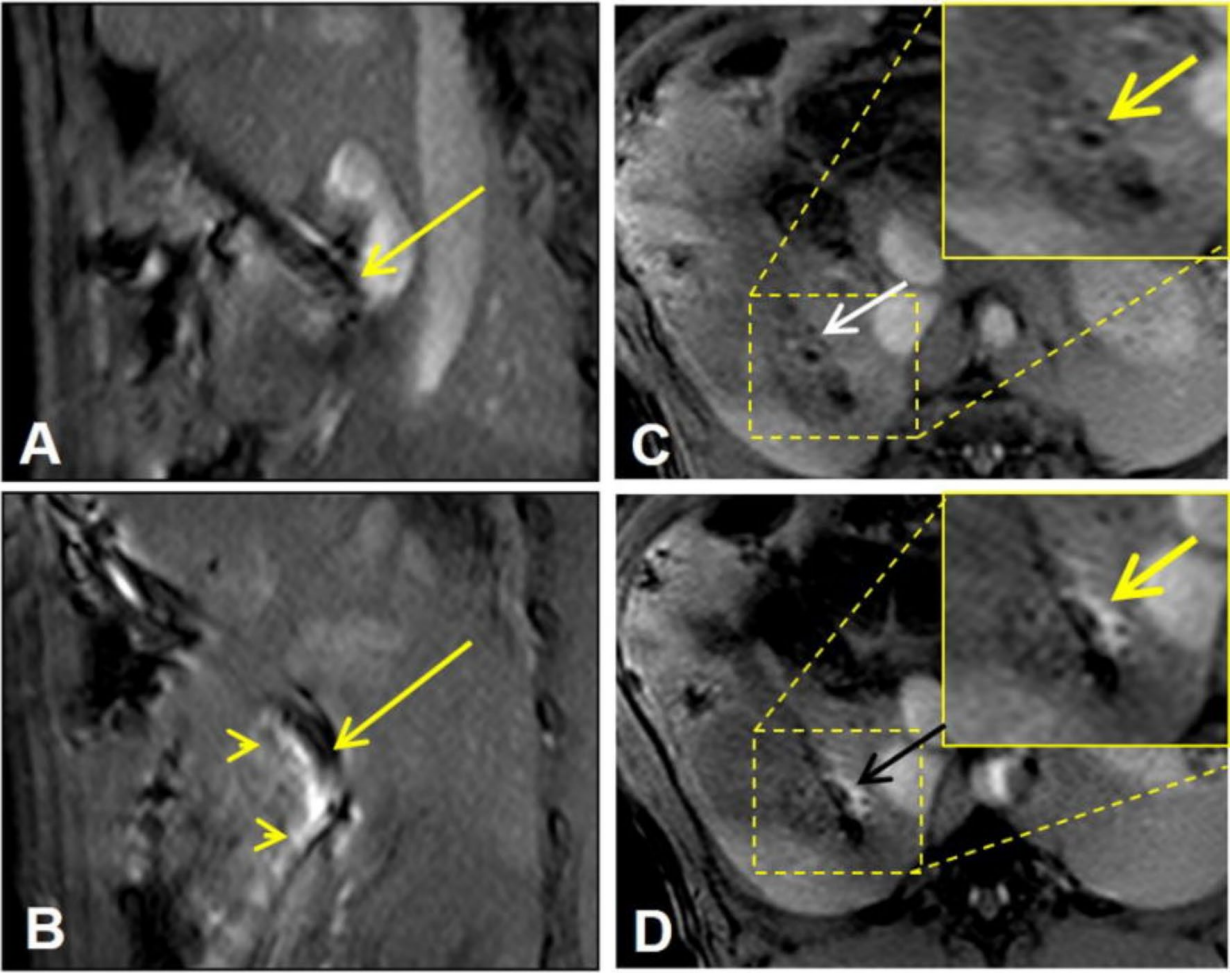
Under the guidance of magnetic resonance imaging, the mixture including motexafin gadolinium was delivered into the choledochus wall of swine. **A** T1 weighted sagittal image; **B** the needle pricked into the choledochus wall (arrow) where motexafin gadolinium was delivered; this was displayed as a high signal interpenetrating the choledochus (arrowheads). **C**, **D** Cross-sectional images of the choledochus before (**C**) and after injection (**D**). Reproduced from Zhang et al. [76]

National Cancer Care Network (NCCN) guidelines recommend CT or MRI for the diagnosis and staging of CCA. Both contrast-enhanced CT and MR scan can identify mass-forming CAA; however, periductal-infiltrating CCA and primary sclerosing cholangitis are difficult to detect by contrast-enhanced CT or MRI. FDG-PET is the most accurate method for systemic staging of CCA, including detection of lymph node stage and distant metastases, as well as for the diagnosis of recurrent disease. However, FDG-PET faces similar challenges to contrast-enhanced CT or MR imaging in differentiating benign from malignant bile duct strictures. This is because increased FDG uptake also occurs in benign bile duct strictures due to secondary inflammation caused by stent placement or primary sclerosing cholangitis [68, 69]. Kim and colleagues [70] reported that FDG-PET, CT, and MR had a diagnostic accuracy of 86%, 68%, and 57% for CCA tumor detection, respectively. The emergence of PET/MR bimodal imaging technology has enabled functional molecular multi-parameter imaging. Although substantial data on the effect of FDG-PET/MR imaging are lacking, empirical evidence suggests that monitoring the biological characteristics of tumors using molecular probes has advantages over PET/CT or MR alone [60]. The maximum standardized uptake value (SUVmax) of primary focus in PET/MR of CCA patients is similar to the SUVmax in PET/CT. For hilar areas with a complex anatomical structure, PET/MR combined with functional information obtained by FDG-PET and the contrast resolution of MR are more effective than PET/CT in assessing the degree of catheter involvement. In a retrospective analysis of 37 patients with CCA, Ferrone et al. [71] used conventional imaging and showed that 15 patients had early resectable disease and 22 patients had advanced disease outside the range of surgical resection. PET/MR changed the clinical management of 11/37 patients (29.7%): in five patients (13.5%), surgery was canceled because of other diseases, and four patients (10.8%) who were “inoperable” underwent surgery. The surgical plan of two patients (5.4%) was changed based on PET/MR. This suggested that PET/MR may influence the management of untreated CCA patients. An added benefit is that patients receive less radiation. PET/MR also has some disadvantages, such as long examination time, and the need to correct the attenuation of SUVmax and other data before it can be used for reference [72, 73]. However, PET/MR is a new nuclear medicine hybrid technology that can improve the local and systemic staging of CCA patients, potentially affecting their clinical treatment [71]. Although molecular imaging has had a great impact on diagnostic imaging, it has not been integrated into the intervention process until recently. Interventional molecular imaging aims to make full use of the advantages of the two imaging fields. Interventional radiology can expand the ability of existing molecular imaging techniques as follows: (a) by reaching deep targets, (b) by allowing careful observation of small targets, (c) by accurately guiding the delivery of non-target imaging tracers or therapies, and (d) by improving the effectiveness of targeted imaging and therapy [74]. In CCA, interventional molecular imaging can be used to monitor the delivery of non-targeted imaging tracers or therapeutic agents to their specific targets. One example is the use of high spatial resolution magnetic resonance imaging to monitor drug delivery to the bile duct wall. Motexafin gadolinium (MGd) is a broad-spectrum anticancer drug. Because of its unique chemical structure, it can function as a radiotherapy and chemotherapy sensitizer as well as facilitating T1-weighted image enhancement of MR images and red fluorescence emission [75]. Zhang et al. locally delivered a mixture of MGd, 5-fluorouracil, and trypan blue into the choledochus wall of swine followed by MR imaging. The results showed that the distribution of MGd in the choledochus wall was clearly displayed by MR imaging (Fig. 6) [76].

**Fig. 7:**
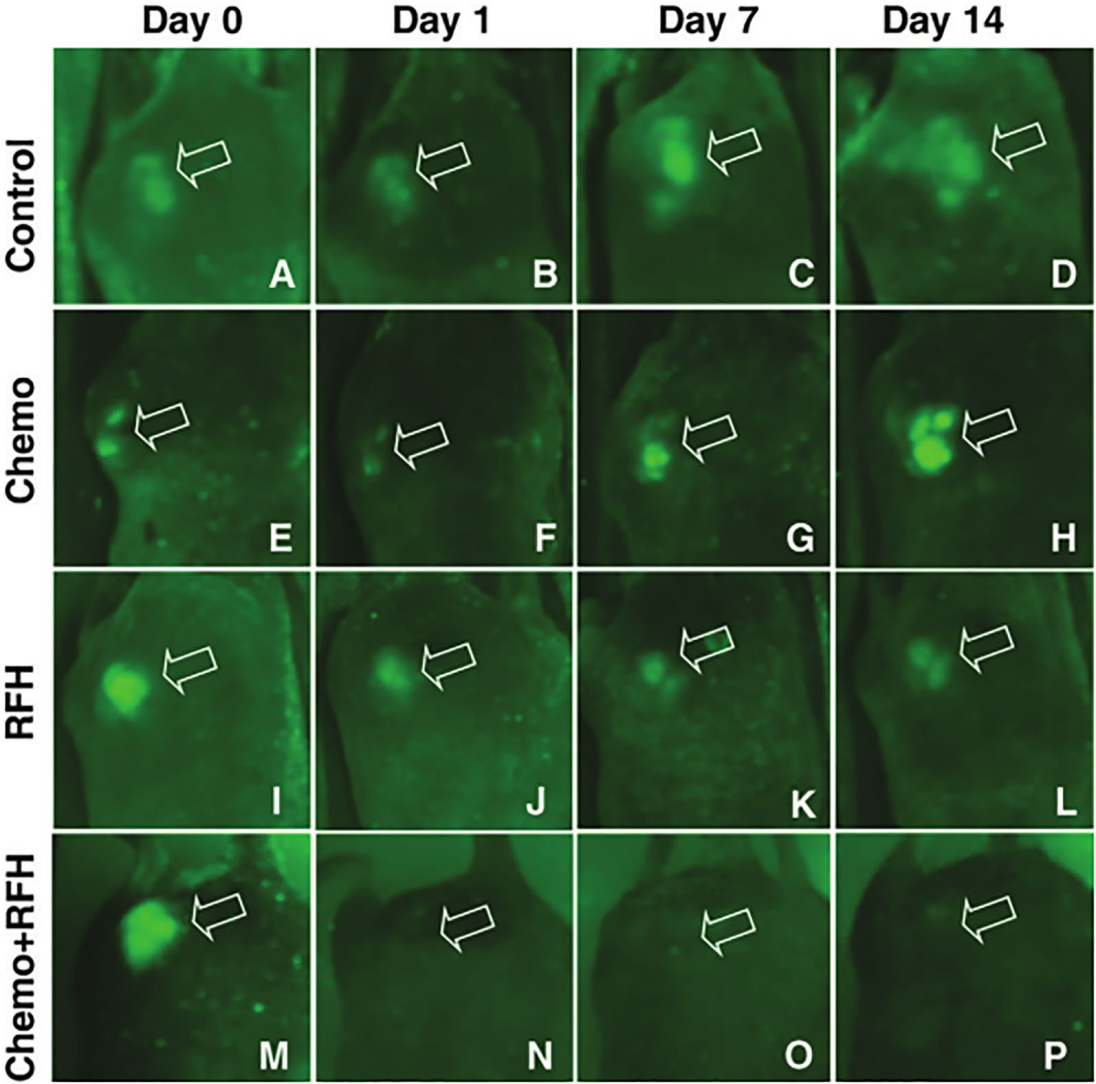
Optical imaging of cholangiocarcinoma lumps (arrow) with different treatments. **A**–**D** Control group; **E**–**H** chemotherapy group; **I**–**L** radiofrequency hyperthermia group; **M**–**P** combined treatment group of chemotherapy and radiofrequency hyperthermia. Reproduced from Zhang et al. [77]

Zhang et al. also studied radiofrequency hyperthermia in the bile duct using an MR heating guidewire to enhance the therapeutic effect of chemotherapeutic drugs on CCA. In the study, they used MR and optical bimodal imaging for monitoring. The results showed that combination treatment with chemotherapy and radiofrequency hyperthermia caused an immediate and significant decrease in the ADC value and the fluorescence signal of the tumor (Fig. 7) [77]. These results provided a theoretical basis as well as a new idea for imaging-mediated monitoring after combined treatment of late CCA.

## Conclusions and future perspectives

CCA is a highly aggressive and heterogeneous malignancy with a dismal prognosis. Despite extensive research, current treatment strategies remain limited and ineffective in most cases. Medical imaging is an important method for the diagnosis and treatment of patients, which is a prerequisite for accurate medicine. The purpose of molecular imaging is to improve the sensitivity and specificity of traditional image detection. The molecular imaging study of CCA is still in the early stages and mostly limited to animal experiments and preclinical research. Thus, there are many difficulties and challenges ahead. The selection of a reliable target to distinguish CCA from other tumors or normal tissues is essential for molecular imaging of CCA. Accordingly, there are currently few mature probes used in CCA molecular imaging. On the one hand, developing or discover probe complexes for CCA targeting is expensive and time-consuming. On the other hand, the safety, specificity, and sensitivity of molecular probes used in the human body need to be further verified. For these reasons, the development of molecular probe complexes for specific monitoring of tumor cell growth and death has become the focus of CCA molecular imaging research. Currently, there is an activatable probe known as “smart probe” that can change the molecular conformation independently according to the microenvironment (such as pH, ion concentration, the partial pressure of oxygen), thus affecting the strength of imaging signals. This is the most advanced tumor imaging probe, and the improved signal-to-noise ratio makes it a potentially effective probe for the molecular imaging of CCA [78].

This review focuses on four different molecular imaging modalities. The current advantages and limitations of each method should be considered when designing future studies involving molecular imaging. PET and SPECT imaging are the earliest molecular imaging modalities used in clinical practice; they are sensitive for the detection of CCA and play a vital role in tumor grading and recurrence monitoring. However, nuclear medicine has poor spatial resolution; therefore, PET and SPECT should be combined with CT or MRI for multimodal imaging in practical applications. MRI has several advantages such as high spatial resolution, excellent soft tissue contrast, and no radiation exposure. Since MRI uses various sequences for imaging, it can be used in CCA to assess the extent of tumor invasion, providing differential diagnosis and prognostic information. Although MRI is a useful imaging method, it has low sensitivity and a long acquisition time. In MR molecular imaging, the unique characteristics of molecular imaging such as high sensitivity are combined to make MRI one of the best molecular imaging modalities. Optical imaging has been widely used in many cell and animal studies in the past few decades. Despite several advantages such as high sensitivity, no radiation, and low cost, the clinical application of optical imaging remains uncertain because of limitations such as light attenuation with increased tissue depth. Endoscopic technology and intraoperative real-time dynamic detection are the main directions for the application of optical imaging to the clinical diagnosis and treatment of CCA. Finally, the multimodal nanomolecular imaging platform can integrate the advantages of these imaging modalities and compensate for the corresponding shortcomings. These techniques increase the diversity of CCA molecular imaging, thus greatly improving the diagnostic accuracy and prognosis of CCA patients.

There are still many new opportunities in CCA molecular imaging research. Molecular imaging research of CCA, which is part of personalized medicine, is moving in the direction of miniaturization of targeted materials, nanoscale imaging media, and multi-modal imaging methods. The translation of animal and preclinical trials to clinical application is the next focus of molecular imaging. In the future, the use of molecular imaging for early screening will greatly benefit patients with CCA.

## Data Availability

All data are published within the manuscript.

## Abbreviations

[^123^I]IOFA: [^123^I]iodooctyl fenbufen amide
^18^F-FDG: Fluorine-18 fluorodeoxyglucose
ADC: Apparent diffusion coefficient
BLI: Bioluminescence imaging
CCA: Cholangiocarcinoma
COX: Cyclooxygenase
CT: Computed tomography
CXCR4: C-X-C motif chemokine receptor 4
DCCA: Distal cholangiocarcinoma
DOT: Diffuse optical tomography
ERCP: Endoscopic retrograde cholangiopancreatography
FLI: Fluorescent imaging
FMT: Fluorescent molecular tomography
GLUT: Glucose transporter
HCC: Hepatocellular carcinoma
hTfR: Human transferrin receptor
ICCA: Intrahepatic cholangiocarcinoma
IL: Interleukin
MGd: Motexafin gadolinium
MR: Magnetic resonance
MRCP: Magnetic resonance cholangiopancreatography
NSAIDs: Nonsteroidal anti-inflammatory drugs
PA: Photoacoustic
PCCA: Perihilar cholangiocarcinoma
pCLE: Probe-based confocal laser endomicroscopy
PET: Positron emission tomography
PTC: Percutaneous transhepatic cholangiography
SPECT: Single-photon emission computed tomography
SPIO: Superparamagnetic iron oxide
SUVmax: Maximum standardized uptake value
TGF: Transforming growth factor
TNF: Tumor necrosis factor
UCL: Upconversion luminescent
US: Ultrasound
VEGF: Vascular endothelial growth factor
